# Responses of isolated balsam-fir stem segments to exogenous ACC, IAA, and IBA

**DOI:** 10.48130/forres-0024-0030

**Published:** 2024-09-30

**Authors:** Rodney Arthur Savidge

**Affiliations:** Independent researcher (retired professor), Fredericton, New Brunswick, E3B 4M6, Canada

**Keywords:** Auxin, ACC, *Abies balsamea*, Leader growth, Intercellular spaces, Cortex, Resin duct, Woody duct, Xylogenesis, Leaf trace

## Abstract

In this investigation, the effects of exogenous indole-3-acetic acid (IAA), indole-3-butyric acid (IBA), and 1-aminocyclopropane-1-carboxylic acid (ACC) on anatomical development within cultured segments of *Abies balsamea* (L.) Mill. were compared, using debudded and defoliated leaders produced in the preceding year as bioassay material. In stem apical regions, IAA promoted radial enlargement of pre-existing cortical resin ducts and attending parenchyma enlargement, whereas IBA promoted cell division and expansion of parenchyma on the outer edge of phloem without altering cortical duct shape. Cortical woody ducts, each partially surrounded by cambium, were observed as a novel but infrequent feature. A single cortical woody duct was spatially associated with each mature leaf as its vascular trace, and they were not encountered elsewhere in the cortex, nor were they induced to form in response to any hormone application. An unknown leaf factor induces the development of cortical woody ducts. Both IAA and IBA promoted cell division in the vascular cambium. The common cellular response at the interface between the latewood boundary and cambial zone was the radial expansion of primary-walled fusiform cambial cell derivatives with little if any ensuing tracheary element (TE) differentiation. Enhanced TE production at basal stem positions occurred when ACC was provided with IAA and/or IBA, and an IAA + IBA + ACC combination produced a basal stem response similar to that in untreated segments having intact leaves. The data support the conclusion that IAA, IBA, and ACC have distinct but complementary roles in the overall regulation of the types of cellular differentiation that contribute to cortex histogenesis and diameter growth of balsam-fir leaders.

## Introduction

*Arabidopsis thaliana* L. ('arabidopsis') and *Populus* ('poplar' and 'aspen') spp. are evolutionarily distantly removed from conifers but, as annual seed plants, have nevertheless become models for explaining plant growth and development^[1,2]^. Arabidopsis is likely inadequate for revealing all mechanisms of secondary growth in trees, because it does not produce annual layers of vascular and peridermal tissues, such as those found in woody species of the temperate zones^[3]^. However, arabidopsis plants can produce some secondary vascular tissue within their flowering stems and roots^[1,4,5] ^ and *Populus* spp. share with arabidopsis many regulatory genes^[2,5]^. There is growing evidence that organogenesis, histogenesis and cellular differentiation in conifers may involve regulatory genes in common with those eudicots^[6]^, although important differences remain to be resolved^[7]^.

Arabidopsis investigations revealed that indole-3-acetic acid (IAA) and indole-3-butyric acid (IBA) have distinct regulatory roles^[8−11]^ and are transmitted in different pathways through plant tissues^[12−14]^. These findings have yet to be confirmed in *Populus* spp., but IBA is effective at inducing rooting of poplar cuttings^[15]^.

The ethylene precursor 1-aminocyclopropane-1-carboxylic acid (ACC) has a variety of roles in regulating arabidopsis and poplar development^[16−18]^, and many interactions between auxin and ethylene have been described^[19−21]^.

In balsam fir (*Abies balsamea* (L.) Mill.), research has indicated that IAA^[22−24]^, ACC^[25,26]^, and ethylene^[27−31]^ have roles as endogenous regulators. IBA as an endogenous hormone has been largely overlooked in conifers, although a full-scan mass spectrum established its presence in the cambial tissue of *Pinus contorta* Dougl. long ago^[32]^, and exogenous IBA applied to pine stem segments promoted xylogenesis although weakly relative to IAA^[33]^. Given the above-described advances with eudicots, this investigation aimed to discover what, if any, effects on secondary growth in balsam-fir leaders might occur in response to IAA, IBA, ACC, and combinations thereof.

Before the balsam-fir segments used in this investigation were treated with hormones, they were debudded and defoliated, so that their only available reserves were those stored within stem tissues. To avoid the well known memory effects induced by gravity on tilted stems and branches^[34,35]^, the stem segments were prepared exclusively from zenith-grown main-stem leaders. After culturing treated stem segments for four weeks under identical conditions, microscopy data were produced and are presented below.

## Materials and methods

### Stem segments of balsam fir (*Abies balsamea* (L.) Mill.)

**Figure 1:**
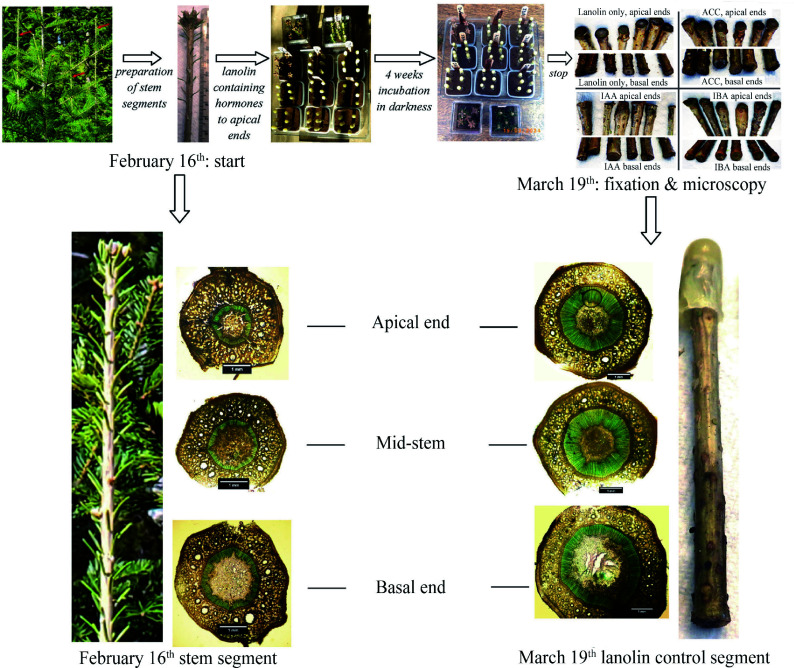
Flowchart showing the progression of the investigation and examples of stem anatomy at the beginning and end. The stem segment at lower left shows stem diameters below the apical whorl of buds, and the segment at lower right shows diameters at the base of the leader.

On Feb. 16^th^, dormant main-stem leaders between 24 and 36 cm in length and produced during the previous year's growing season were collected (Fig. 1). The leaders had no obvious defects and displayed perfectly zenith growth (i.e., negative geotropism). The source trees were a wild-type population of saplings, between 1−2 m in height, growing in wild forest at Fredericton, New Brunswick, Canada (45°55'38.56"N, 66°37'54.18"W).

Not all stem segments survive an extended incubation period after having been debudded and defoliated^[32]^. The explanation for this variability remains uncertain, but with the aim of having at least three replicates for analysis, six stem segments were used for each treatment.

Using a razor blade, leaders were subdivided under cold tap water into 9-cm-long segments and, except where otherwise noted, all leaves and buds were removed by careful razor-blade trimming. Leaves were cut away through the petiole region just above their circular attachment pads. The terminal bud cluster was fully removed by a transverse stem cut at its base, and axillary buds were sliced off tangentially flush with the epidermis.

To know how intact buds and leaves influenced diameter growth, three additional treatments were prepared: segments having both buds and leaves; segments having leaves but with buds removed; and segments with buds intact and leaves removed.

The segments prepared as described above were inserted basal ends down, to a depth of 3.5 cm, into plastic containers (7 cm × 7 cm) containing prewashed Oasis 5,200 horticube foam bricks (Smithers Oasis, Kent, OH, USA 44240). Water to saturate the foam was added to the surface level of the foam and replenished regularly as needed. Six segments were inserted into each container. From the leader's terminal bud whorl to the branch internode below, stems increase slightly in diameter. Pre-examination of eight dormant leaders at several distances below the apical buds revealed no obvious difference in anatomy. Figure 1 illustrates this size difference by showing the stem near the apical whorl of buds on Feb. 16^th^ and a stem segment taken from a more basal leader position on March 19^th^. Stem segments used in the various treatments were selected randomly from the total pool of stem segments.

### Applications of hormones in lanolin paste

IAA (Sigma-Aldrich Chemicals I3750) was dissolved in absolute ethanol, and IBA-K^+^ (Sigma-Aldrich Chemicals I7152) and ACC (Sigma-Aldrich Chemicals A3903) were dissolved in distilled H_2_O to known concentrations. Appropriate volumetric aliquots of those concentrated solutions, together with additional ethanol and/or H_2_O were transferred into 50 g of warm (70 °C) liquid lanolin (Fisher L7-500), sealed and shaken thoroughly, to produce lanolin mixtures having 1.0 mL of ethanol and 1.0 mL of H_2_O. The pastes as so prepared contained 3.0 μmols of each test compound per gram of lanolin. The 'control' paste was 50 g of lanolin that had been supplemented with 1.0 mL of ethanol and 1.0 mL of H_2_O.

After thorough mixing, 1.0 ± 0.1 g of warm liquid lanolin was transferred into a pre-weighted gelatin half-capsule, cooled to solidify and the half-capsule upended over the apical end of a stem segment (Fig. 1), repeating the process for each stem segment.

The experiment was incubated at a daytime temperature of 22 °C, gradually declining after 12 h to 15 °C and climbing back again daily to 22 °C. All stem segments treated with lanolin paste were shielded from light using a loosely fitted cover of aluminum foil.

Stem segments not provided lanolin were incubated in light. The photoperiod was 16 h using natural daylight illumination supplemented with 60 W incandescent lighting after sunset.

After a treatment period extending from Feb. 16^th^ to March 19^th^, the stem segments were transferred into methanol until sectioned for microscopy (Fig. 1).

### Bright-field microscopy

After rinsing off surface methanol, each stem segment was sectioned at three positions (Fig. 1): 1 cm below the apical end, 1 cm above the basal end, and halfway between the two ends. Both transverse and radial sections were cut by hand-held razor blade from the two ends, and transverse sections only were cut from the mid-stem region. Sections were stained for 30 s in 0.05% (w/v) aqueous (unbuffered) toluidine blue O^[36]^, rinsed in distilled water and examined by bright-field microscopy. Photomicroscopy images were obtained at 32× and 500× magnifications.

Overview (32× magnification) analysis of cross sections was used to assess if there was evidence for circumferential variation in a stem's cambial response, but such variation was mostly lacking. Cell types outward from the latewood boundary were counted in six adjoining radial files, distinguishing tracheids (TEs), radially expanded thin-walled cells centripetal to the cambial zone (RE), fusiform cambial cells of the cambial zone (CZ), and enlarging and enlarged phloem cells (Ph) centrifugal to the CZ^[37,38]^. The average number per radial file of each cell type was recorded.

New TEs produced in response to a treatment could be confidently distinguished by reference to the latewood boundary, also by their thinner secondary walls, larger radial diameters, and darker secondary-wall staining than those of preexisting latewood. Cambial derivatives having actively lignifying (blue-green stained) secondary walls were included in the TE count. When uncertain by examination of cross sections if a radially expanded thin-walled cambial derivative was undergoing lignification, radial sections were examined to search for evidence for bordered-pit formation and, when developing bordered pits were found, the cell was considered a TE, whereas if lacking the cell was counted as a radially expanded primary-walled (RE) derivative.

Radial file counts of phloem cells (Ph) included all cells adjoining and centrifugal to the cambial zone to the cortex boundary that displayed no signs of radial compression. The counts do not necessarily comprise newly produced phloem cells; some probably pre-existed in the dormant state.

Linear measurements of photomicroscopy images were done using ImageJ software^[39]^ and a 1.00 mm (10 μm divisions) stage micrometer as a calibration standard.

### Scanning electron microscopy

SEM preparation and analyses were done using critical point drying (acetone - CO_2_) and carbon coating using SEM aquisition conditions described previously^[40]^.

### Statistical analysis

Calculations of means, standard deviations, and unpaired Welch t tests, assuming normal distributions, were done using Microsoft Excel and an alpha of 0.05 to generate *p* values on the basis of unequal variance, as was indicated by the data^[41,42]^.

### Electronic supplemental information

In addition to the findings reported herein, Supplemental Table S1 and Supplemental Fig. S1 in support of the methods and results are available online.

## Results

### Macro responses

After 28 d, at the termination of the experimental trial period, visual examination and microscopy data indicated that only four stem segments of some treatments had apical, mid-stem and basal tissues that had all survived. For example, two that had been provided IAA showed no apical swelling, and they displayed cortical shrinkage responses in their lower regions (Fig. 1). Viewed under the microscope, their internal tissues were brown and evidently dead; therefore, they were rejected.

Segments treated with either IAA or IBA either individually or in combination displayed relatively strong diameter growth to produce swollen stem apices. Stem swelling was most pronounced at approximately 1 cm below the apical end of the stem segment, and though tapering basally was evident for an axial distance of 2−3 cm below the application site (Fig. 1). Lanolin and ACC resulted in little if any apical swelling, but slight basal swelling occurred in response to ACC. Basal swelling was more evident in response to treatments that combined IAA or IBA with ACC, and basal swelling was most obvious on segments having both buds and leaves intact (see Supplemental Fig. S1).

### Microscopy results – dormant stems

At the start of the investigation, the vascular cambium was dormant. First periderm formation was evident at scattered locations around circumferences. Common throughout the cortex were vertical files of small parenchyma cells, some circular and others rectangular in outline, with diameters between 5 and 20 μm. Transverse diameters of cortical resin ducts were found to be between 50 μm and > 200 μm in diameter. Each duct was surrounded by two to three tiers of small-diameter sheath cells. Ducts were conspicuously distributed throughout the cortex, spaced 200 μm or more apart within smaller diameter parenchyma. Cortical ducts having the largest diameters appeared well spaced apart around a circumference occupying a position approximately 1/3 the transverse radial distance from the outermost phloem to the epidermis (Fig. 1, see Feb. 16^th^). Cortical ducts of smaller diameters were abundantly present both on the inside and outside of that circumferential zone of larger ducts. In the interfacial region between outermost phloem cells and cortical tissue, incompletely developed presumably nascent resin ducts appeared as enlarged thin-walled parenchyma cells having diameters between 50 and 100 μm and lacking one or more of the tiers of circumferentially bounding parenchyma that are associated with mature resin ducts.

In addition to resin ducts and adjoining parenchyma cells, there were within the dormant cortex aerenchyma surrounded by intercellular spaces. Rarely seen were elongated fibres, some of which were thin-walled though many displayed secondary walls that stained blue-green with toluidine blue. Some but not all parenchyma cells also yielded similar histochemical evidence for lignin or suberin. Widely spaced polyphenolic parenchyma were present but rare in the cortex; they were more commonly associated with the phloem.

### Microscopy results – treated stem segments

**Figure 2:**
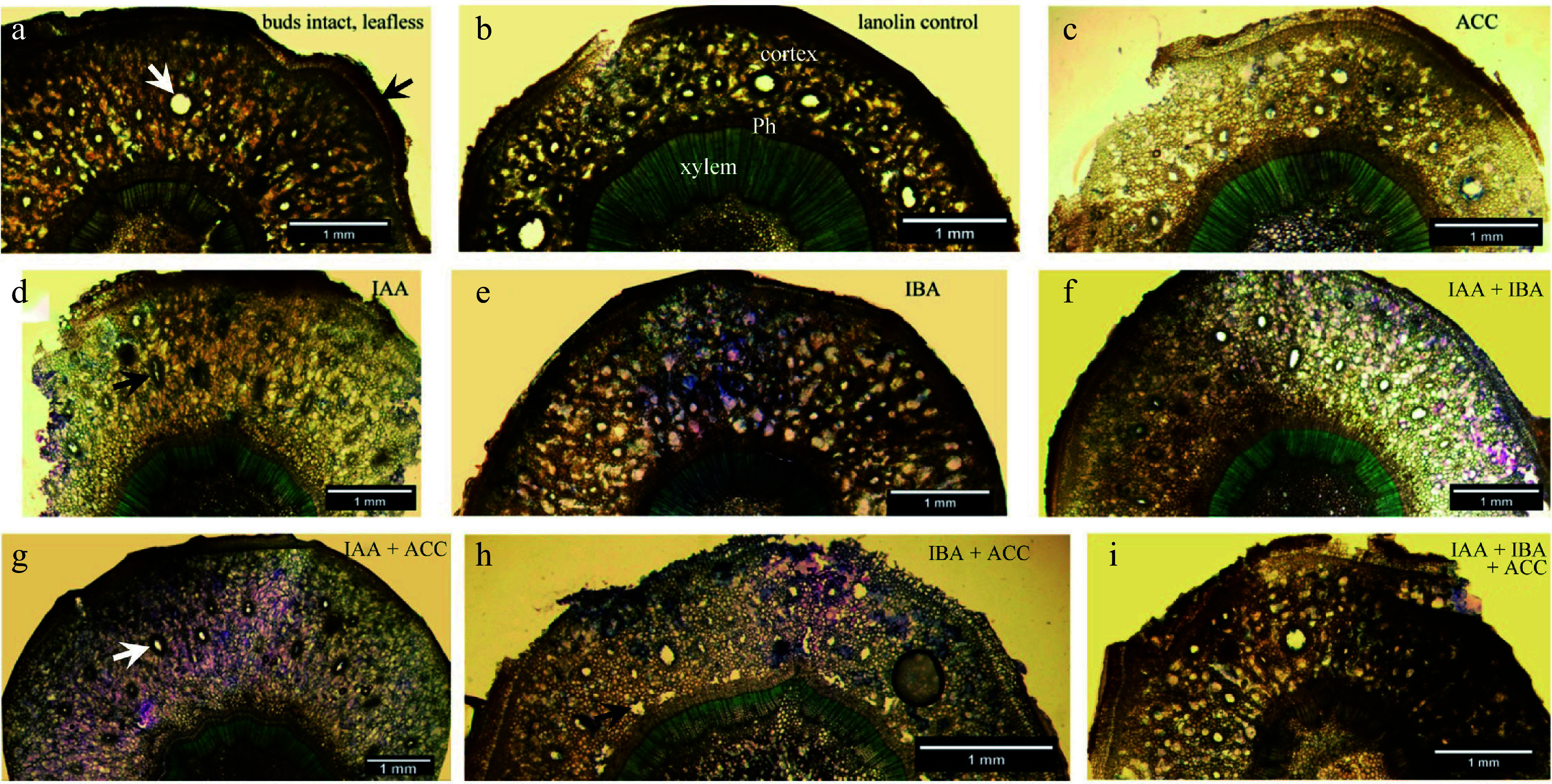
Overview cross sections of stem segments at an apical position approximately 1 cm below the hormone application site. The text at upper right in each photomicrograph indicates the treatment. (a) Black arrow points to evidence for the first periderm enveloping the cortex; the white arrow points to an enlarged cortical resin duct; numerous small ones are also evident. (b) Labels indicate cortex, phloem (Ph) and secondary xylem (xylem) locations. (c) Thinner section than those shown in Fig. 2a & b but otherwise similar. (d) Parenchyma have proliferated and preexisting resin ducts are enlarged or stretched radially. (e) Note the diffuse porosity throughout the cortex and the band of large diameter resin ducts immediately centrifugal to the phloem. (f) Radial enlargement of preexisting resin ducts and proliferation of cortical parenchyma, but lacking is an external band of new resin ducts just beyond the phloem. (g) Preexisting resin ducts are radially enlarged (arrow). (h) Circumferential band of resin ducts has developed external to the phloem. (i) A similar response to that shown in Fig. 2e.

**Figure 3:**
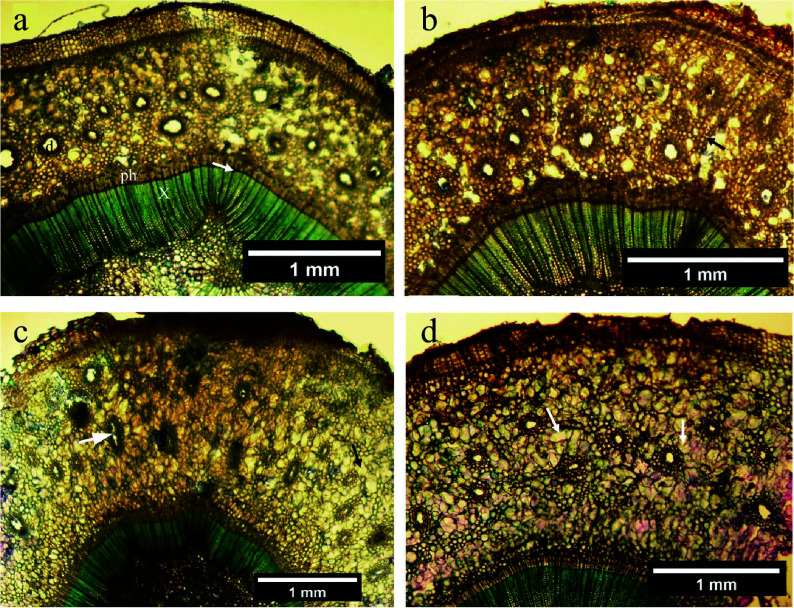
Cross sectional anatomy of the cortex surrounding secondary xylem (staining blue green) at approximately 1 cm below the apical ends of stem segments. (a) Lanolin control, showing cortical resin ducts (d), mature phloem (ph) and secondary xylem (x). The arrow indicates dormant vascular cambium. (b) ACC treatment, showing evidence for browning (arrow) of secondary cell walls in the cortex and phloem. (c) IAA treatment, radially elongated ducts (white arrow) and greatly enlarged parenchyma (black arrow). (d) IBA treatment, numerous enlarged parenchyma (white arrows); ducts retained their circular appearance.

**Figure 4:**
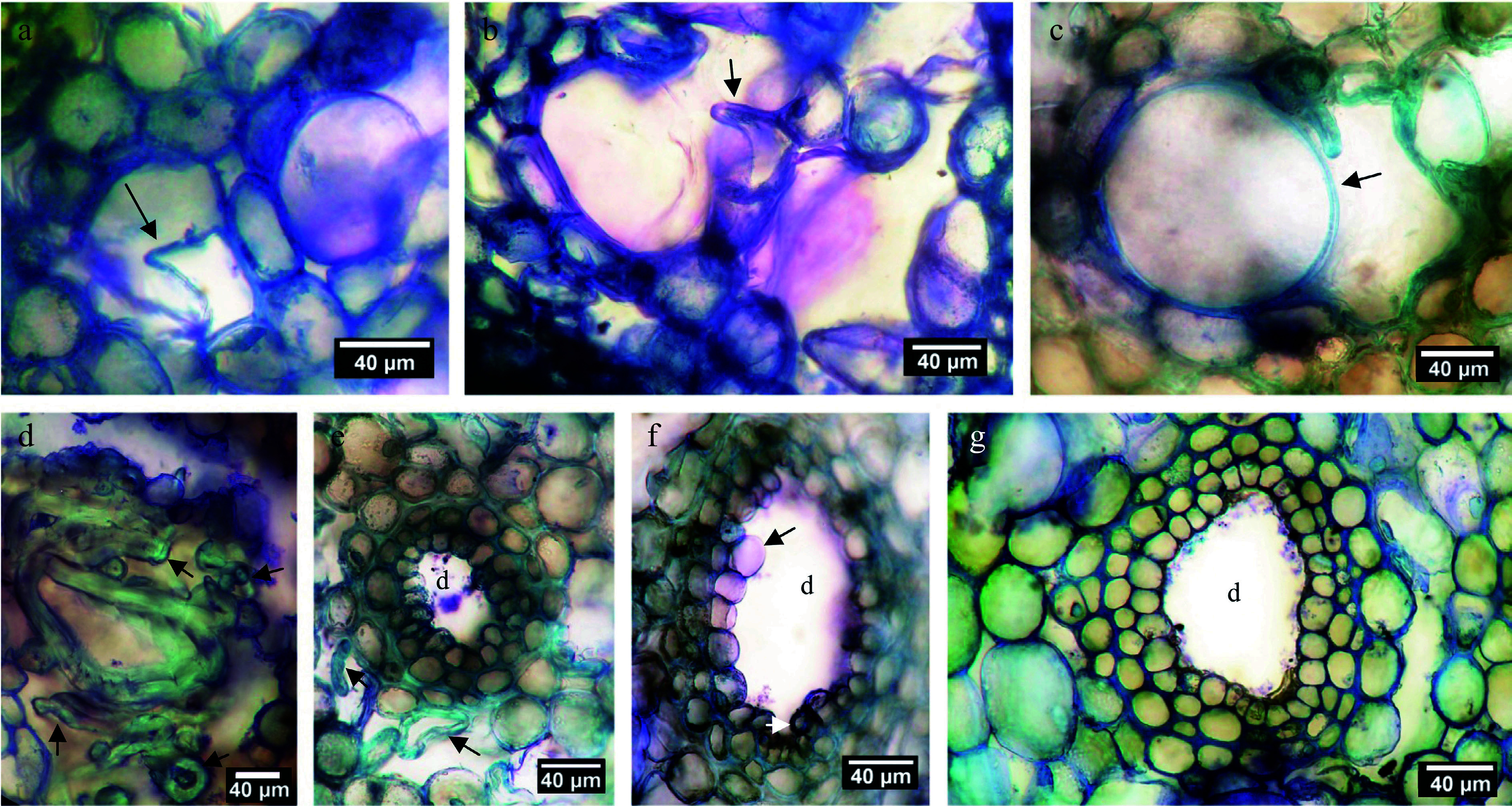
Higher magnification images of cortical tissue. (a) Early stage of cortical duct formation near the outer phloem interface; the wall of a plasmolysing cell is arrowed. (b) Primary wall (arrowed) fully collapsed. (c) Tonoplast (arrowed) of an expanded vacuole in a greatly enlarged cell. (d) Walls of several collapsed cells in the vicinity of a partially collapsed are arrowed; note wall thickness and the staining reaction possibly indicative of lignin or suberin. (e) An early duct (d) surrounded by two tiers of sheath cells with collapsed and collapsing cells (arrowed) nearby. (f) Active production of cells (one arrowed) to produce the inner sheath tier surrounding a duct (d). (g) A duct (d) surrounded by three tiers of sheath cells having contents probably indicative of resin production.

**Figure 5:**
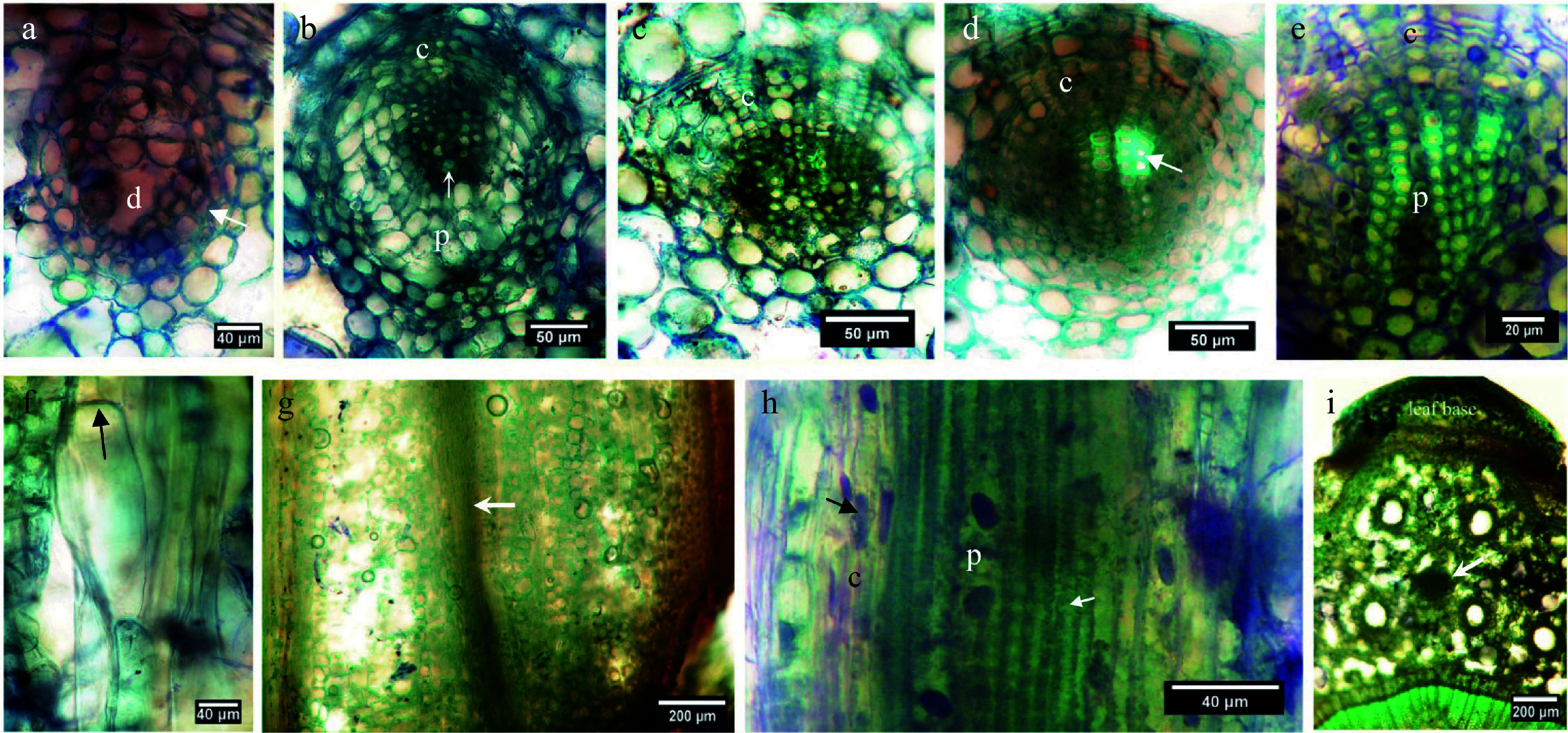
Woody duct formation as observed in cross sections (a)−(e) and (i), and radial longitudinal sections (f)−(h) of balsam fir cortex. (a) Early stage of a developing woody duct (d) encased by variable number of parenchyma tiers (arrow) and most of the duct opening filled with intrusive parenchyma. (b) A slightly more advanced stage with the former duct opening completely filled with parenchyma, some differentiating woody elements, and nascent cambium (c) developing on its outer periphery. The arrow indicates a ray-like string of enlarged parenchyma cells bisecting the woody element population. (c) A more advanced stage of cambium (c) formation in a developing woody duct. (d) The cambium (c) in this woody duct is fully developed but the woody elements appear to be at different stages of secondary wall formation (arrow). (e) A fully mature woody duct bisected by a radial file of parenchyma cells (p). (f) An intrusive tip (arrow) of a parenchyma cell elongating within a cortical duct. (g) Low magnification, showing a longitudinal strand (arrow) containing woody elements and non-woody parenchyma and running axially through the cortex. (h) A longitudinal section through a cortical woody duct showing its cambium (c) with elongated nuclei (black arrow) and internalized parenchyma (p). The duct's woody elements all appear to have annular ribs of the primary xylem type (white arrow). (i) This low magnification cross section through the circular attachment pad of a mature leaf shows the spatial association of the leaf base to a woody duct (arrow).

**Figure 6:**
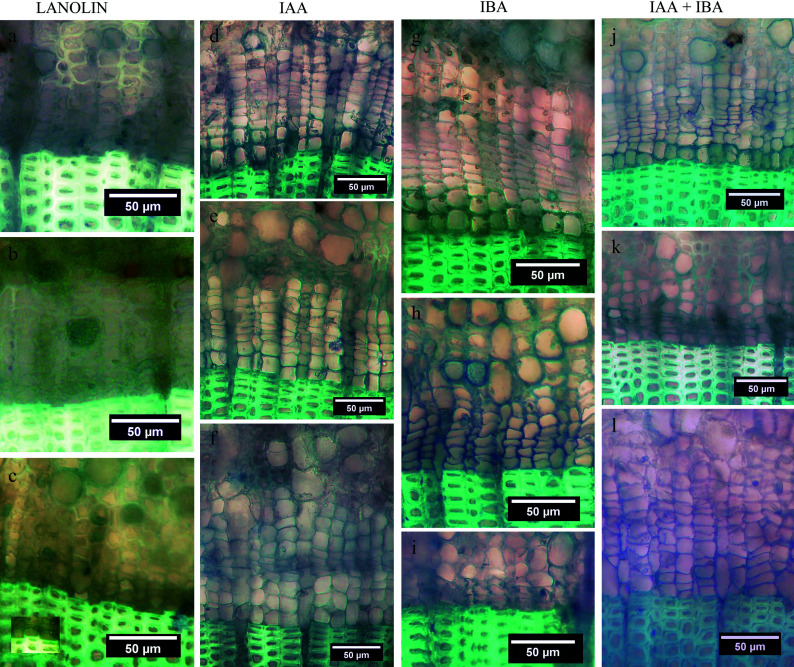
Developmental responses, as seen in cross sections, of stem segments the apical ends of which were treated with lanolin (only), IAA in lanolin, IBA in lanolin, and a combination of IAA and IBA in lanolin. In each of the four columns of photomicrographs, the apical end is shown at the top, followed by the mid-stem region, and the basal end at the bottom. Lanolin column: (a) Non-dividing CZ, several phloem cells per radial file; (b) non-dividing CZ; (c) same as Fig. 6a but with 2−3 RE cells and, as shown in the inset, in scattered locations around the circumference a single TE per radial file. IAA column: (d) new TEs; (e) a single RE and a single thin-walled TE per radial file; (f) RE cells without TEs. IBA column: (g) new TEs; (h) RE only; (i) no RE or TE cells. IAA + IBA column: (j) new TEs; (k) RE cells only; (l) RE cells only.

**Figure 7:**
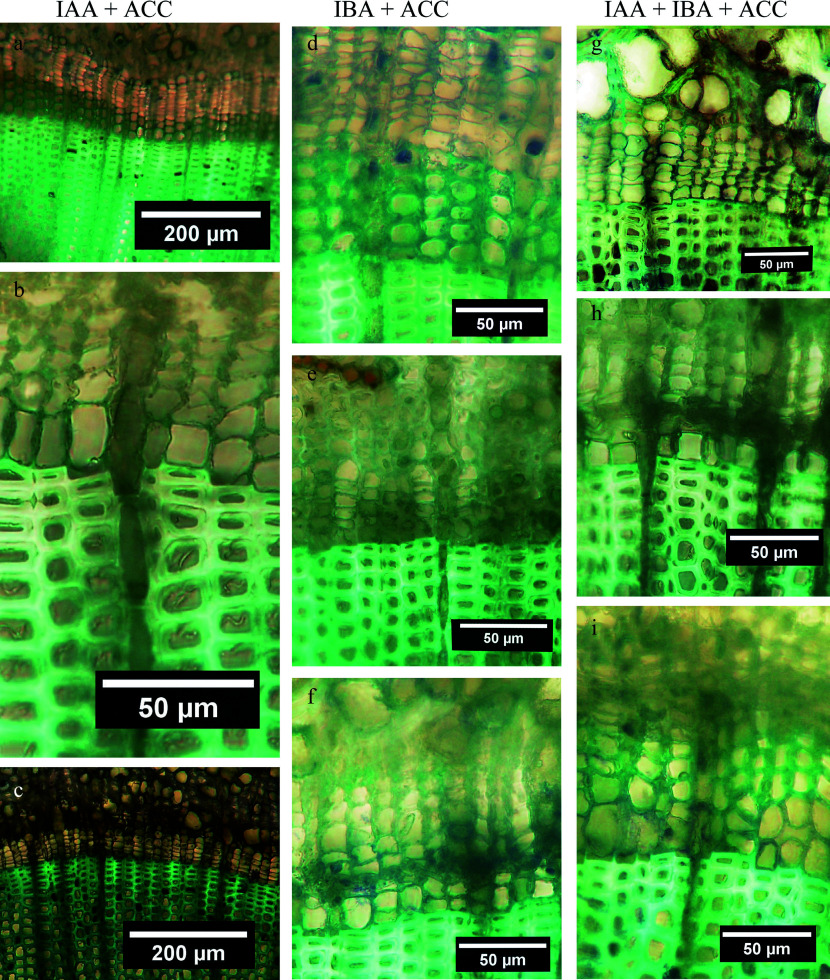
Examples of xylogenic responses, as viewed in cross sections, of stem segments the apical ends of which were treated with a combination of IAA + ACC in lanolin, IBA + ACC in lanolin, and IAA + IBA + ACC in lanolin. In each of the three columns of photomicrographs, the apical end is shown at the top, followed by the mid-stem region, and the basal end at the bottom. IAA + ACC column: (a) Several new TEs per radial file; (b) 1−2 TEs; (c) 1−2 RE cells only per radial file. IBA + ACC column: (d) several new TEs; (e) 1−2 RE cells and no TEs; (f) a single RE cell, 1−2 new TEs and a very narrow CZ. IAA + IBA + ACC column: (g) a single RE cell and no new TEs; (h) a narrow CZ and a single TE; (i) several new TE cells per radial file.

Low magnification cross sections of the apical regions of the stem segments show the three tissue systems (cortical, vascular, pith) present (Figs 2, 3). Higher magnification images are provided in Figs 4−7.

Compared to the strong cortical growth that resulted in swelling at the apical ends of stem segments in response to all treatments involving IAA or IBA (Fig. 1), the vascular cambium's contribution to radial growth in those same stem segments was weak (Table 1), not apparent in Fig. 2 and barely visible in Fig. 3. The pith showed no evidence of any response to any treatment.

**Table 1:** Microscopy data and P values (95% confidence) summarized for the eight treatments.

Treatment	Mean number of cells per radial file																
	Apical end						Midstem						Basal end				
**lanolin**																	
Replicate	Ph	CZ	RE	SL	Total		Ph	CZ	RE	SL	Total		Ph	CZ	RE	SL	Total
1	3	4	1	0	8		4	4	1	0	9		5	5	1	0	11
2	4	5	1	1	11		5	4	1	0	10		4	6	1	2	13
3	4	6	0	0	10		3	5	1	0	9		3	4	2	0	9
4	2	4	0	0	6		4	6	0	0	10		3	4	1	0	8
Mean	3.3	4.8	0.5	0.3	8.8		4.0	4.8	0.8	0.0	9.5		3.8	4.8	1.3	0.5	10.3
Std. Dev.	1.0	1.0	0.6	0.5	2.2		0.8	1.0	0.5	0.0	0.6		1.0	1.0	0.5	1.0	2.2
**ACC**																	
Replicate	Ph	CZ	RE	SL	Total		Ph	CZ	RE	SL	Total		Ph	CZ	RE	SL	Total
1	4	4	2	0	10		2	6	0	0	8		4	6	1	2	13
2	3	4	2	0	9		3	5	0	0	8		3	5	2	1	11
3	4	4	1	1	10		4	4	1	0	9		3	4	2	0	9
4	3	5	0	0	8		5	5	1	0	11		4	4	2	0	10
Mean	3.5	4.3	1.3	0.3	9.3		3.5	5.0	0.5	0.0	9.0		3.5	4.8	1.8	0.8	10.8
Std. Dev.	0.6	0.5	1.0	0.5	1.0		1.3	0.8	0.6	0.0	1.4		0.6	1.0	0.5	1.0	1.7
P: ACC vs lanolin	0.337	0.201	0.119	0.500	0.350		0.271	0.353	0.269	1.000	0.274		0.337	0.500	0.104	0.365	0.367
**IAA**																	
Replicate	Ph	CZ	RE	SL	Total		Ph	CZ	RE	SL	Total		Ph	CZ	RE	SL	Total
1	5	6	5	3	19		5	4	1	2	12		3	4	2	1	10
2	4	4	1	3	12		2	2	2	0	6		3	0	4	0	7
3	2	6	3	2	13		3	6	1	1	11		4	6	3	0	13
4	2	4	2	3	11		3	4	1	2	10		2	2	4	0	8
Mean	3.3	5.0	2.8	2.8	13.8		3.3	4.0	1.3	1.3	9.8		3.0	3.0	3.3	0.3	9.5
Std. Dev.	1.5	1.2	1.7	0.5	3.6		1.3	1.6	0.5	1.0	2.6		0.8	2.6	1.0	0.5	2.6
P: IAA vs lanolin	0.500	0.375	0.036	0.000	0.032		0.181	0.233	0.104	0.040	0.432		0.140	0.138	0.008	0.338	0.340
P: IAA vs ACC	0.386	0.149	0.095	0.000	0.042		0.395	0.165	0.049	0.040	0.319		0.180	0.138	0.022	0.201	0.231
**IBA**																	
Replicate	Ph	CZ	RE	SL	Total		Ph	CZ	RE	SL	Total		Ph	CZ	RE	SL	Total
1	4	8	4	2	18		6	4	2	0	12		3	6	0	0	9
2	5	6	2	1	14		5	2	2	1	10		3	2	2	0	7
3	2	4	1	2	9		2	2	3	0	7		2	2	2	0	6
4	2	4	1	1	8		2	6	2	0	10		2	2	2	0	6
Mean	3.3	5.5	2.0	1.5	12.3		3.8	3.5	2.3	0.3	9.8		2.5	3.0	1.5	0.0	7.0
Std. Dev.	1.5	1.9	1.4	0.6	4.6		2.1	1.9	0.5	0.5	2.1		0.6	2.0	1.0	0.0	1.4
P: IBA vs lanolin	0.500	0.259	0.061	0.009	0.120		0.416	0.151	0.003	0.196	0.414		0.038	0.092	0.338	0.196	0.028
P: IBA vs ACC	0.386	0.143	0.209	0.009	0.144		0.423	0.111	0.002	0.196	0.287		0.025	0.092	0.338	0.108	0.008
P:IBA vs IAA	0.500	0.337	0.262	0.009	0.314		0.348	0.353	0.015	0.065	0.500		0.180	0.500	0.022	0.196	0.081
**IAA + ACC**																	
Replicate	Ph	CZ	RE	SL	Total		Ph	CZ	RE	SL	Total		Ph	CZ	RE	SL	Total
1	3	7	3	1	14		2	5	3	0	10		3	7	3	2	15
2	4	8	2	1	15		3	6	2	2	13		3	8	3	1	15
3	3	3	4	2	12		2	4	2	0	8		2	6	1	3	12
4	2	6	2	3	13		1	4	2	2	9		2	5	0	0	7
Mean	3.0	6.0	2.8	1.8	13.5		2.0	4.8	2.3	1.0	10.0		2.5	6.5	1.8	1.5	12.3
Std. Dev.	0.8	2.2	1.0	1.0	1.3		0.8	1.0	0.5	1.2	2.2		0.6	1.3	1.5	1.3	3.8
P: IAA + ACC vs lanolin	0.353	0.174	0.005	0.022	0.007		0.007	0.500	0.003	0.091	0.341		0.038	0.038	0.282	0.135	0.202
P: IAA + ACC vs ACC	0.180	0.102	0.034	0.022	0.001		0.053	0.353	0.002	0.091	0.236		0.025	0.038	0.500	0.195	0.254
P: IAA + ACC vs IAA	0.391	0.227	0.500	0.065	0.451		0.077	0.233	0.015	0.375	0.444		0.180	0.033	0.076	0.074	0.141
P: IAA + ACC vs IBA	0.391	0.371	0.209	0.337	0.318		0.096	0.151	0.500	0.149	0.436		0.500	0.016	0.396	0.051	0.031
**IBA + ACC**																	
Replicate	Ph	CZ	RE	SL	Total		Ph	CZ	RE	SL	Total		Ph	CZ	RE	SL	Total
1	5	6	1	5	17		3	5	1	0	9		5	4	1	2	12
2	6	4	2	2	14		3	4	1	2	10		5	4	2	1	12
3	3	3	1	2	9		3	4	1	2	10		2	2	1	2	7
4	6	8	3	5	22		4	6	1	0	11		4	2	3	0	9
Mean	5.0	5.3	1.8	3.5	15.5		3.3	4.8	1.0	1.0	10.0		4.0	3.0	1.8	1.3	10.0
Std. Dev.	1.4	2.2	1.0	1.7	5.4		0.5	1.0	0.0	1.2	0.8		1.4	1.2	1.0	1.0	2.4
P: IBA + ACC vs lanolin	0.046	0.350	0.038	0.014	0.042		0.089	0.500	0.196	0.091	0.180		0.390	0.030	0.201	0.160	0.442
P: IBA + ACC vs ACC	0.061	0.219	0.244	0.014	0.052		0.368	0.353	0.091	0.091	0.139		0.274	0.030	0.500	0.244	0.318
P: IBA + ACC vs IAA	0.070	0.425	0.178	0.229	0.307		0.500	0.233	0.196	0.375	0.433		0.139	0.500	0.034	0.065	0.395
P: IBA + ACC vs IBA	0.070	0.435	0.390	0.050	0.200		0.333	0.151	0.008	0.149	0.416		0.061	0.500	0.365	0.040	0.045
P: IBA + ACC vs IAA + ACC	0.030	0.323	0.095	0.071	0.261		0.024	0.500	0.008	0.500	0.500		0.061	0.003	0.500	0.384	0.181
**IAA + IBA**																	
Replicate	Ph	CZ	RE	SL	Total		Ph	CZ	RE	SL	Total		Ph	CZ	RE	SL	Total
1	2	2	4	2	10		4	4	1	0	9		5	6	4	0	15
2	2	2	2	2	8		3	6	1	2	12		1	4	4	0	9
3	2	2	2	2	8		3	8	1	3	15		3	8	1	3	15
4	3	2	2	1	8		2	5	3	2	12		2	6	1	1	10
Mean	2.3	2.0	2.5	1.8	8.5		3.0	5.8	1.5	1.8	12.0		2.8	6.0	2.5	1.0	12.3
Std. Dev.	0.5	0.0	1.0	0.5	1.0		0.8	1.7	1.0	1.3	2.4		1.7	1.6	1.7	1.4	3.2
P: IAA + IBA vs lanolin	0.065	0.005	0.010	0.003	0.423		0.067	0.178	0.122	0.034	0.066		0.178	0.123	0.124	0.293	0.174
P: IAA + IBA vs ACC	0.009	0.001	0.061	0.003	0.160		0.271	0.235	0.073	0.034	0.045		0.228	0.123	0.229	0.390	0.225
P: IAA + IBA vs IAA	0.140	0.007	0.405	0.015	0.028		0.376	0.095	0.338	0.276	0.129		0.402	0.053	0.242	0.189	0.118
P: IAA + IBA vs IBA	0.140	0.018	0.293	0.269	0.102		0.268	0.065	0.122	0.046	0.105		0.398	0.030	0.183	0.126	0.019
P: IAA + IBA vs IAA + ACC	0.089	0.017	0.365	0.500	0.001		0.067	0.178	0.122	0.207	0.134		0.398	0.324	0.269	0.310	0.500
P: IAA + IBA vs IBA + ACC	0.012	0.030	0.160	0.067	0.040		0.312	0.178	0.196	0.207	0.101		0.152	0.014	0.242	0.390	0.155
**IAA + IBA + ACC**																	
Replicate	Ph	CZ	RE	SL	Total		Ph	CZ	RE	SL	Total		Ph	CZ	RE	SL	Total
1	2	5	2	0	9		5	6	1	1	13		3	6	1	5	15
2	5	8	1	3	17		4	2	2	0	8		4	4	1	2	11
3	3	6	3	0	12		5	3	3	0	11		4	8	1	3	16
4	4	2	2	3	11		3	4	3	0	10		4	2	2	1	9
Mean	3.5	5.3	2.0	1.5	12.3		4.3	3.8	2.3	0.3	10.5		3.8	5.0	1.3	2.8	12.8
Std. Dev.	1.3	2.5	0.8	1.7	3.4		1.0	1.7	1.0	0.5	2.1		0.5	2.6	0.5	1.7	3.3
P:IAA + IBA + ACC vs lanolin	0.384	0.364	0.014	0.124	0.072		0.353	0.178	0.022	0.196	0.207		0.500	0.433	0.500	0.037	0.131
P: IAA + IBA + ACC vs ACC	0.500	0.243	0.140	0.124	0.088		0.195	0.126	0.013	0.196	0.142		0.269	0.433	0.104	0.050	0.168
P: IAA + IBA + ACC vs IAA	0.405	0.432	0.235	0.124	0.283		0.128	0.420	0.065	0.065	0.336		0.089	0.158	0.008	0.028	0.089
P: IAA + IBA + ACC vs IBA	0.405	0.440	0.500	0.500	0.500		0.341	0.426	0.500	0.500	0.313		0.009	0.135	0.338	0.024	0.016
P: IAA + IBA + ACC vs IAA + ACC	0.271	0.333	0.140	0.406	0.266		0.006	0.178	0.500	0.149	0.375		0.009	0.176	0.282	0.145	0.424
P: IAA + IBA + ACC vs IBA + ACC	0.084	0.500	0.353	0.077	0.179		0.065	0.178	0.040	0.149	0.339		0.378	0.114	0.201	0.095	0.117
P: IAA + IBA + ACC vs IAA + IBA	0.074	0.040	0.235	0.399	0.056		0.048	0.074	0.160	0.046	0.194		0.166	0.271	0.124	0.084	0.418
Legend (t tests): values in green, *p* ≤ 0.05; values in yellow, *p* = 0.05−0.10; values without any color, *p* > 0.10.																	

In stem segments of all treatments, axial resin ducts circumscribed by tiers of sheath cells were present (Fig. 3e−g) throughout the cortex. As already noted for dormant stems, the duct opening appeared to be at several stages of ontogeny from phloem to epidermis.

The cortex in stem segments treated with lanolin (Fig. 3a) or ACC (Fig. 3b) appeared similar and unchanged from cortex anatomy as viewed at the start of the investigation. It appears possible that ACC somewhat enhanced cortical aerenchyma development and altered secondary wall thickness and chemistry of pre-existing parenchyma (Fig. 3b); however, those aspects remain for future investigation.

In swollen apical regions of stem segments treated with IAA, cortical ducts became greatly stretched radially, possibly in response to radial enlargement of adjoining parenchyma connected through cell-wall bonds to the ducts. Many cortical parenchyma cells underwent expansive primary-wall growth in response to IAA treatment, but no evidence was found for development of new resin ducts (Figs 2d, 3c).

In swollen apical regions of stem segments treated with IBA, preexisting cortical tissue became altered into a more diffuse tissue comprising exceptionally large primary-walled cells with numerous intercellular gaps (Figs 2e, 3d). A pinkish-colored reaction of primary cell walls to toluidine blue (Fig. 3d) is evidence of recently produced cell walls, probably due to strong radial expansion in pre-existing cell walls that enveloped small diameter parenchyma cells. In two of the four examined stem segments treated with IBA, the cortex displayed a phloem-enveloping zone of enlarged cortical resin ducts (see also Fig. 2e, h), but as can be seen in Fig. 3d this zone was not invariably present, and therefore it could not be decided if they were newly formed in response to the IBA treatment.

Figure 4 shows several aspects of cortical resin duct formation as observed in stem segments that served as controls to the phytohormone treatments. There was abundant evidence for cell division, expansion, making and breaking of intercellular bonds, and cellular differentiation (Fig. 4a−g). Collapsed cells and intercellular spaces were present throughout (Fig. 4a−e). Plasmolysis occurred in thin-walled parenchyma (Fig. 4a, b), but thereafter those collapsed cells developed thickened walls (Fig. 4d). Non-collapsed primary-walled parenchyma in the vicinity of collapsed cells became greatly enlarged, then separated schizogeneously (Fig. 4a−c), followed within developing ducts by what appeared to be lysigeneous bursting (Fig. 4c−g).

Circumferential tiers of parenchyma cells were produced around the duct by control of the cell-division plane (Fig. 4f). Cells at the exterior surface of the duct sheath enlarged and produced thickened secondary cell walls in the tiers of the sheath border (Fig. 4e−g). With advanced duct development, those tiers of circumferential sheath cells changed biochemically (compare Fig. 3a & g), presumably in support of resin formation and secretion.

In addition to resin ducts, cortical woody ducts were present as a novel anatomical feature (Fig. 5a−e). Examination of cross sections of 26 randomly selected distantly spaced stem positions from eight stem segments fixed on the starting day of the experiment yielded only nine woody ducts in a total cortical resin duct population estimated at > 1,500. After the March 19^th^ conclusion of the investigation, woody ducts were found present in lanolin control and all hormone treatments, but no convincing evidence was observed for any experimental treatment having either increased their frequency or altered their diameter.

Additional investigation of dormant stem segments collected and fixed in winter revealed an invariable spatial association of cortical woody ducts with mature leaf attachment points to the stem (Fig. 5i). Depending on their stage of development, woody duct transverse diameters were determined to be between 80 μm and 200 μm. Investigation of dormant stem segments collected and fixed in winter revealed an invariable and obligate spatial association of cortical woody ducts with mature leaf attachment points to the stem (Fig. 5i). Woody ducts extended axially through the cortex for at least 1 mm (Fig. 5g, h). Over that longitudinal distance, some portions of the woody duct displayed non-woody parenchyma while other axial positions in the same duct displayed woody elements (Fig. 5g), possibly explainable in terms of varied extents of woody duct maturation at different axial positions (Fig. 5a−e).

Cortical woody ducts basal to mature leaves were initiated through similar processes described above for cortical resin ducts (Fig. 4), but as the woody duct channel opened up it became occupied by extending parenchyma cells (Fig. 5a, b, f) that subsequently divided to create ordered radial files of elongated small-diameter cells within the duct followed by the production of annular ribs of lignified secondary walls similar to those of primary xylem (Fig. 5b−e, h). No bordered pits were seen in the woody elements. Ray-like nucleated parenchyma cells, uniseriate and rarely bi- or tri-seriate, sub-divided the radial files of woody elements of each cortical woody duct; these duct-bisecting parenchyma evidently emerged early in woody duct formation (Fig. 5a−c). As with vascular tissues, files of woody elements displayed radial polarity.

Also associated with cortical woody ducts and not with other resin ducts of the cortex was an arc of cambium-like cells having elongated nuclei. These cambial cells arose within or near the radially external border of the outermost tier of the sheath cells surrounding the developing woody duct (Fig. 5b−e, h). Formation of the woody-duct cambium was not essential for the cells that differentiated into woody elements within the duct to be produced, as the cambium formed after, rather than before, the appearance of the duct's woody cells (Fig. 5a, b).

### Microscopy results - phloem and xylem development

Supplemental Table S1 provides radial file cell number data for dormant stem segments at the start of the experiment.

After the 28-d experimental treatment, no evidence was found for traumatic resin canals (TRC) on the inner (centripetal) side of the vascular cambium having been produced as a response to any hormone treatment. Debudded stem segments having intact leaves also displayed no TRCs. In contrast, segments having only intact buds and those having both buds and leaves produced readily detectable TRCs in the first formed earlywood.

Table 1 presents each of the four analyzed stem segments at its three examined positions cell counts per radial file (averages based on six radial files) of the several cell types observed. Means, standard deviations, and *p* values are provided. Green boxes indicate results whereby the Welch t test it is 95% or more probable that the null hypothesis should be rejected. Yellow boxes in Table 1 have *p* values between 0.05 and 0.10, too high to reject the null hypothesis but possibly an indication that if a larger sample size had been tested, rejection may have been indicated.

Tests comparing stem-segment positions and cell types support the interpretation of qualitative differences seen during microscopy examinations. IAA and IBA promoted some xylogenesis in the apical portions of the stem segments, but the response at lower positions was limited to radial expansion of primary-walled cambial derivatives; they did not become TEs despite the 28-d incubation period. TEs differentiated at all three stem-segment positions in only one of the four segments provided IAA, and in none of those provided IBA.

IAA + IBA in combination yielded increased radial file cell numbers in both the CZ and the zone of earlywood TEs, particularly in the apical region of stem segments; however, only two of the four segments produced new TEs in the basal region. A similar result was produced by the IAA + ACC combination. In contrast, the IAA + IBA + ACC treatment favored xylogenesis primarily in the basal region of stem segments, with very little TE production occurring above (Table 1).

Examples of stem-segment responses to lanolin, IAA, IBA and a combination of IAA + IBA are provided in Fig. 6. Protoplasm of vascular cambium in segments receiving lanolin alone (Fig. 6a−c) cleared from its previously condensed dormant state, but otherwise there was no change in three of the four stem segments. The fourth lanolin control segment produced one new TE per radial file in two locations, only, around the entire circumference of the apical region, and two TEs per radial file were present but only at one circumferential location in the basal region (see Fig. 6c inset). In the mid-stem of that same segment, the cambial zone remained unproductive and adjoining the latewood.

IAA had its greater TE-inducing effect at apical ends (Fig. 6d), weaker at mid-stem regions (Fig. 6e) and absent in three of the four segments at basal ends (Fig. 6f, Table 1). IBA also promoted TE differentiation at apical ends (Fig. 4g) but not at lower stem-segment positions (Fig. 6h, i, Table 1). The response to an IAA + IBA combination in two of the four stem segments was similar to that of IBA alone (Fig. 6j−l); however, the other two stem segments displayed new xylem at each of the three examined positions. IAA + IBA clearly had a positive effect on the number of cells per radial file in the cambial zone (Table 1).

Figure 7 provides examples of how stem segments responded to auxin + ACC mixtures. IAA + ACC elicited a relatively strong xylogenic response at apical ends (Fig. 7a), weaker at mid-stems (Fig. 7b) and strongest at stem bases in three of the segments (Table 1), but the fourth failed to respond in a like manner (Fig. 7c). IBA + ACC produced xylogenic results similar to those of IAA + ACC (Fig. 7d−f; Table 1). The triplet combination of IAA + IBA + ACC promoted xylogenesis in basal locations but in only one of the four mid-stem positions and in only two of the four apical regions (Fig. 7g−i; Table 1).

**Figure 8:**
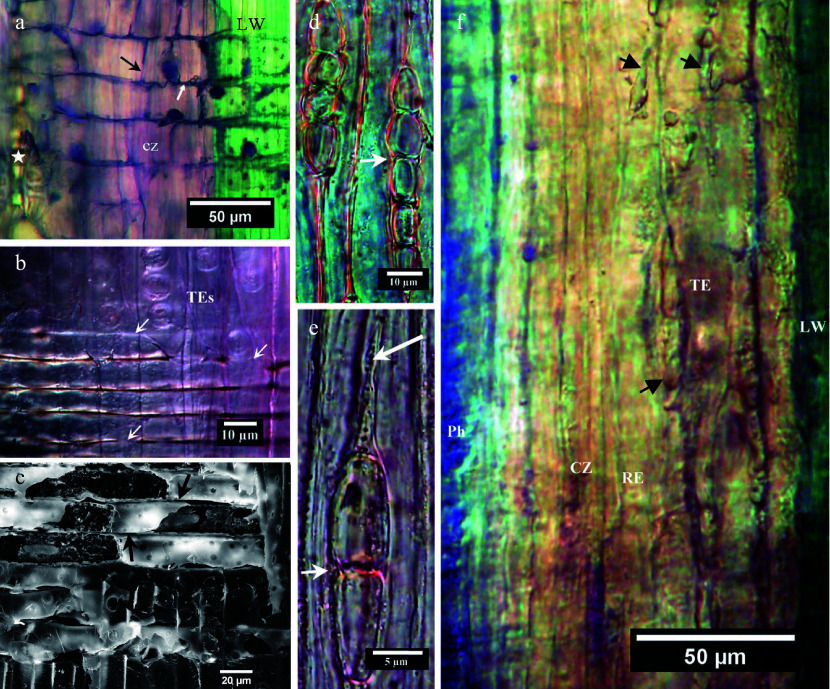
Sections of balsam-fir cambium and xylem that display variable darkening of intercellular spaces. (a) Brightfield radial section of cambial zone (cz) bordering latewood (LW) and stained with toluidine blue. The white arrow indicates a darkened compound middle lamella region and the black arrow an absence of similar darkening. (b) The white arrows indicate intercellular spaces between procumbent xylem ray cells that stained less intensively to toluidine blue. (c) SEM of a xylem ray in radial section; the arrows point to intercellar spaces between the procumbent ray cells. (d) Tangential section showing two rays on the centripetal periphery of the cambial zone. The arrow points to an intercellular space between the radial wall of a fusiform cell and that of a ray cell. (e) Tangential section of a 2-celled ray in the cambial zone, the small arrow pointing to evidence for an intercellular space between fusiform and ray cell walls. An axially oriented 'spear tip' (large arrow) appears to contain particulate matter and to be intruding between what had been adjoining walls of two fusiform cells. (f) Radial section (interference contrast optics) of phloem (Ph), cambial zone (CZ), radially expanding cambial derivative (RE) and differentiating TEs in proximity to latewood (LW). The arrows point to accumulations of insoluble matter within or paralleling the axial walls.

It was observed in radial sections of the vascular cambium region that ACC, both by itself and in combination with an auxin, enhanced the dark coloration of coarse cell-wall-adjoining lines visible between axial and radial elements. These mostly horizontal radial 'lines' are intercellular spaces between procumbent ray cell walls (Fig. 8a, b). Although narrow, the spaces are evident when viewed in the SEM (Fig. 8c).

The dark coloration within intercellular spaces when viewed by brightfield microscopy extended radially along procumbent rays from mature xylem through cambial zone into mature phloem, although in the cambial zone the staining reaction to toluidine blue, a metachromatic dye, was different from that in phloem and xylem, indicating chemical differences (Fig. 8a). Figure 8d & e are tangential sections viewed by differential interference contrast optics and showing the triangular shapes of the intercellular spaces in the cambial zone. The large arrow in Fig. 8e points to a spearhead-shaped intrusion between walls of two fusiform cells, perhaps related to the deposits of material associated with the axial walls of fusiform cells at an early stage of TE differentiation (Fig. 8f).

Data for those stem segments cultured in this experiment but not treated with a lanolin paste are provided in Supplemental Table S1 and Supplemental Fig. S1.

## Discussion

The primary focus of this investigation was to discover if qualitative differences in tissue anatomy might emerge, following applications of ACC, IAA, IBA, and their combinations to the apical ends of balsam-fir stem segments. The working hypothesis based on earlier research was that varied expressions of xylogenesis, e.g., formation of primary vs secondary xylem TEs should be visible after those hormones were applied at identical concentrations dispersed in lanolin. Unexpectedly, differences in cortical development in response to hormone treatment were also found. In addition, several cellular phenotypes, possibly entirely novel, also were observed as natural phenomena of non-treated stems.

The concept of vascular development being regulated principally by auxin originated in herbaceous species^[37]^ and, when applied to conifers, has limitations depending on the age and stage of development of the stem segments investigated^[3]^. Previous investigations with several conifer species revealed that IAA was entirely ineffective in promoting xylogenesis when stem segments older than two years were subjected to the same treatment that stimulated xylogenesis in young stems^[38,43]^. For this reason, only stem segments prepared from leaders that had grown in the preceding year were investigated.

At the start, the cambium in the investigated stem segments was fully dormant; the stems lacked any evidence of cell division or any stage of earlywood formation. Under the bioassay's culture conditions, a four-week incubation period was assumed to be more than ample for cambial reactivation, cell production and completion of cellular differentiation to occur. After stopping the experiment, that assumption was confirmed by observations that earlywood tracheids had matured.

Because all segments receiving hormone treatment were first debudded and defoliated, lacked roots, and were provided only water, the metabolism underlying the observed growth and development responses can be assumed to have drawn upon storage reserves within the cortex, ray, and pith parenchyma cells. Remarkably, almost all segments appeared still healthy after 28 d, and the few having any evidence of dead cambium were rejected.

Although the experimental timeframe proved adequate for newly produced cambial derivatives to become fully differentiated, evidence for TE differentiation was present at only some, not all, of the three examined stem-segment positions, even in treatments where xylogenesis appeared to be more strongly induced. Within stem segments displaying earlywood TEs, examination of three positions (apical, basal, and mid-stem) revealed other positions where no TEs had been produced anywhere around the stem circumference. At those positions, centripetal cambial derivatives were enlarged but remained primary walled and lacked evidence for initiation of bordered-pit development, the earliest stage indicating cellular commitment to TE differentiation^[40]^.

In relation to phloegenesis, similar radially enlarged cambial derivatives accumulated centrifugal to the cambial zone, presumably as nascent phloem cells. Many of those derivatives on the cambium's centrifugal side were primary-walled, not fully differentiated as either sieve cells or other phloem elements. This incomplete development of phloem was particularly apparent in response to IBA treatments.

Xylogenesis occurred in response to IAA, IBA, and IAA + IBA, but compared to cortical growth responses at the apical ends of those stem segments, all xylogenic responses were relatively weak. Thus, no conclusions are offered about which hormonal treatment was more effective based on radial file cell number counts, mainly because cortical development appeared to be a more favored response but also given the heterogeneous anatomical responses associated with the vascular cambium and its derivative cells. The *p* values (Table 1, Supplemental Table S1) are provided merely as indications of possibly productive avenues of future research.

Observations on the presence/absence of cell types, in particular of TEs, appear to be more important than any deductions that might be inferred based on quantitative data. For example, in two of the four stem segments treated with IAA + ACC, no TEs were produced at their mid-stem region despite apical and basal regions having produced new TEs (Table 1). Earlier papers concerned with hormonal effects in the balsam-fir stem segment bioassays have overlooked this natural variation in responsiveness, perhaps because only single rather than multiple positions in stem segments were observed.

The leaders used to produce stem segments in this investigation were from a single population of sapling trees growing nearby one another on the same site, and all leaders were selected based on being overtly healthy, straight, and of zenith orientation. Based on their past growth performance, it can be imagined that all would have grown similarly strongly in both height and diameter had they remained intact on their source trees and received contributions from roots, leaves, and buds. But again, the three examined positions within each stem segment produced different responses, even those within the lanolin control, and variation among the four replicates further corroborated this intrinsic variability.

Longitudinal variation over lengths of tree trunks in springtime cambial reactivation, the onset of cambial dormancy, and rates of xylem development are established natural processes^[3,37,38]^. With current knowledge, somatic genomic variation at different positions within the stem of the tree cannot be confidently excluded as a plausible explanation for such variation^[3,44]^. However, it seems more probable that the unequal variance revealed by microscopy in this study could be explainable in terms of intrinsic metabolic differences between stem positions and between genotypes, for example in their storage and endogenous hormone reserves, the status of dormancy release or other physiogenetic considerations, in particular, mechanisms of uptake and internal transmission of exogenous hormones.

Differences between stem positions in their xylogenic responsiveness to auxin were earlier noted in other conifer species^[32,33]^. However, those qualitatively different developmental responses had no explanation and were relegated to the realm of artifacts. The observations made in this investigation negate an artifactual interpretation. The relatively small diameters of balsam-fir stem segments enabled complete cross-sectional examination of the cambial region around entire stem circumferences in the microscope. Moreover, the bioassayed stem segments when first collected as leaders were all of the zenith orientation, and the stem segments were subsequently maintained in their vertical orientation throughout the investigation. Therefore, it is assumed that the well-known effect of gravity on inducing circumferentially unequal cambial growth responses was not a complicating factor in this investigation.

The inconsistency of the cambial response over the length of the stem segment seems to indicate that the cambium competence for xylogenesis varies, in terms of the intrinsic ability of cambial cells to receive, transmit, or respond to hormones. The observed heterogeneity indicates the individuality and complexity of the numerous underlying physiological processes that function during cambial growth and xylogenesis^[3]^. Before initiating further experiments, there is a need to know how to better characterize, screen, and standardize the intrinsic variation existing within the specimens selected for investigation. When debudded-defoliated stem segments from leaders are intended as the bioassay specimens, it can be recommended that measures be taken to ensure that all initially are investigated and confirmed to have identical apical and basal diameters and equal numbers and distributions of leaves and buds. In reality, achieving such standardization will not be easy, because casual examination reveals that each individual leader on a tree grows somewhat uniquely from those of neighboring trees. As already noted, there is also need during microscopy to observe full stem circumferences at apical, mid-stem and basal stem-segment positions.

Earlier investigations into balsam-fir stem-segment bioassay responses to hormones, auxin, in particular, have retained mature leaves intact on the stem segments. Young developing leaves have been described as the primary source of IAA in balsam-fir trees^[23]^, but investigations with other conifer species indicated that mature leaves and dormant buds both contributed IAA to cambium^[3,23,32,45]^. In pine species, IAA was exported from mature leaves during winter dormancy as well as during the growing season^[45]^. Past research has also revealed that mature conifer leaves when intact on stem segments effectively promoted xylogenesis^[32,38,43,46−48]^, and the same was again observed in this investigation (Supplemental Table S1).

In the present investigation, buds left on stem segments whether or not defoliated remained dormant throughout the trial period, and cambium nevertheless reactivated and produced a small amount of new xylem. None of the budbreak, shoot elongation, or new leaf development was necessary for the resumption of diameter growth. The regulation of dormancy release in apical and lateral meristems must differ, as the vascular cambium in stem segments reactivated while the shoot apical meristems remained dormant. Similar findings have been noted in other conifer species^[37,38]^.

It is noteworthy that traumatic resin canals were produced in spatiotemporal association with xylogenesis in non-experimental stem segments having buds, and TRCs were not produced in debudded-defoliated stem segments subjected to any of the hormone treatments, despite the razor-blade wounding that was done to remove leaves and buds. Two plausible explanations can be suggested, that buds export a TRC-inducing factor (such as methyljasmonic acid) or, alternatively, that the lanolin carrier used to provide hormones to segments either blocked or absorbed the TRC-inducing factor.

In addition to IAA, mature leaves export many other compounds, possibly including ACC for which there is growing evidence for a role in xylogenesis^[3]^. Exogenous ACC applied by itself to needle stumps on stem segments resulted in xylogenesis independently of preceding cambial cell division or radial expansion of cambial derivatives^[43]^. The inductive effect of ACC was observed to be similar to that of mature leaves, resulting in both cambial fusiform and cambial ray cells transforming into tracheary elements, mostly of primary-xylem cell types having altered fine structure^[48]^ and lacking the bordered pits characteristic of secondary-xylem tracheids^[40]^.

In another investigation, an IAA + ACC combination was more effective than IAA alone at inducing cambial cell division, radial enlargement and differentiation of cambial derivatives into new tracheids^[43]^. Similar complementary and seemingly synergistic effects of auxin and ACC were noted in relation to xylogenesis in *Armoracia rusticana* roots cultivated *in vitro*^[49]^.

In the present investigation, no convincing evidence was found for applied ACC having induced xylogenesis independently, though it appeared to enhance the promotion induced by both IAA and IBA. Plausibly, the present observations can be reconciled with earlier findings on the basis that they showed that little if any ACC oxidation to ethylene occurred when ACC was supplied to cambium cells cultured *in vitro* except when IAA was also provided^[25]^. Because the present investigation began with debudded and defoliated dormant stem segments, it may be that insufficient endogenous IAA was present to promote ethylene production.

Segments having mature leaves, only, yielded the stronger xylogenic response, but only at the basal stem-segment location. Of the several hormone treatments investigated, debudded-defoliated stem segments treated with an IAA + IBA + ACC combination responded most similarly to segments having intact mature leaves (compare Table 1 with Supplemental Table S1). Investigations to quantify how much IAA, IBA, and ACC are present in mature balsam-fir leaves and allocated to vascular development remains to be done.

Small intercellular spaces that are oriented radially between tiers of procumbent ray cells have frequently been considered as transverse pathways for gas exchange^[50−52]^. This investigation has provided evidence that those pathways have continuity from xylem to non-collapsed phloem, possibly extending even into the cortex. It could not be conclusively demonstrated but seems reasonable to suspect that aqueous fluids - those continually present in the secondary xylem, vascular cambium and secondary phloem - would diffuse into those intercellular spaces and traverse those tissues. Some intercellular spaces stained with toluidine blue while others displayed darkened contents. Based on this and observations made in an earlier investigation^[43]^, it can be suggested that these intercellular spaces between procumbent ray cell walls do more than transfer gases radially.

Excepting reactivation of dormant cambium and subsequent development of vascular tissues^[23,24,27−31]^, little knowledge yet exists about how overall stem development occurs during the balsam-fir leader's second year of life. Advances have been made in understanding post-cortical stages of periderm formation in fir trees^[53−57]^. For example, large-diameter parenchyma cells in the bark of *Abies firma* and *A. homolepis,* were observed long ago^[53,54]^. However, descriptions of the cortex associated with the first two years of a fir tree stem's life generally have been brief, portraying a relatively simple tissue system. A prevalent but assumptive concept is that epidermal, cortical, vascular, and pith development of stems during their first year arise as largely predetermined structures within the dormant shoot apical meristem.

This study revealed the balsam-fir leader cortex to be a complex milieu of different cell types, shapes, and evident specializations, an impressive diversity of mostly living cells capable of growing and differentiating. Such complex histogenesis requires regulation; it cannot simply arise through predetermined developments in the apical meristem.

The promotion by auxin treatments of cortical diameter growth, viewed macroscopically as stem swelling, was particularly visible for approximately 3 cm below the apical ends of stem segments (Fig. 1). The cellular expansion activity underlying swelling of the cortex indicates that both IAA and IBA can move through cortical tissues, in the process causing small-diameter primary-walled parenchyma to enlarge into quite large cells. This is an important observation worthy of further research, as there has been a tendency to assume that auxin transmission in stems is restricted to cambium and phloem. The axial continuity and zonation of enlarged cortical parenchyma suggests the possibility that a signal translocated intercellularly through the tissue while still at only the brick stage is the explanation for other cells extending or enlarging radially^[3,38,48]^.

Previous investigations into young arabidopsis seedlings revealed IAA and IBA to move preferentially in the basipetal direction through the hypocotyl, both at similar transport rates (8−10 mm·h^−1^)^[9]^. Nevertheless, the transmission of IAA evidently was facilitated by a different protein complex than that of IBA^[9]^. The arabidopsis hypocotyl is anatomically, morphologically and evolutionarily distant from balsam-fir stem segments, but given the observed differences in cortical responses of balsam-fir stem segments to IAA and IBA, it seems possible that different mechanisms of transmission of those two auxins through cortex might explain the observed differences in cortical development.

On the other hand, IBA has been described as merely a precursor of IAA in arabidopsis and other eudicots^[11,13,14]^. That concept is difficult to reconcile with the distinguishably different growth responses of the cortex of balsam fir in response to IAA and IBA. This seems to be evidence that those two endogenous auxins may not only have different transport requirements but also different regulatory effects in balsam-fir stems.

Some cortical parenchyma responded to IAA and IBA by dividing and enlarging to increase the girth of the cortex. In the case of IAA, radial expansion of parenchyma cells resulted in distortion of preexisting resin-duct shape, as viewed in transverse sections, from rounded to radially extended, but this shape change was not attended by evidence for enlargement of the ducts. The change from a circular to radially extended shape indicates cell-wall bonding between the outer tiers of the resin duct and the adjoining parenchyma, although all around were aerenchyma and intercellular spaces. In the case of IBA, enlarged parenchyma arose proximal to the outermost phloem and gave the impression of being nascent ducts. Other primary-walled parenchyma in the same region and further out in the cortex displayed radial extension growth.

ACC did not appear to affect the anatomy of the cortex, but it did appear to alter parenchyma cell wall thickness and staining reactions to toluidine blue; however, more intensive research is needed to corroborate these provisional interpretations.

Sites of cortical resin duct formation appeared to be where numbers of collapsed cells among enlarged parenchyma were greater. Concomitant plasmolysis and turgid expansion in neighboring cells of the cortical tissue system occurs. If this is the explanation, the aqueous environment is not hypertonic for the general cellular population. Plasmolysis-susceptible cells either have inadequate osmotica or are compromised in their ability to regulate osmotic pressure, relative to their neighbors which, by their swelling, evidence a hypotonic environment. Cellular collapse augments intercellular space formation, but how such changes in the cortex may trigger non-burst living parenchyma cells to produce tiers of sheath cells surrounding enlarged parenchyma cells remain unclear.

IAA has been reported to be a signal for periderm formation in arabidopsis roots^[58]^, but no evidence was seen for that in this investigation. More research is needed, but based on observed cortical duct development, it could be that IBA is part of the explanation. Many additional signaling mechanisms, including wounding and pressure, deserve consideration^[59−61]^.

Sites displaying cortical resin ducts were mainly within longitudinal zones of enlarged parenchyma. However, ducts were absent from similar zones where enlarged parenchyma appeared as schizogeneous aerenchyma^[62]^. As thin-walled parenchyma cells enlarge, some burst thereby producing intercellular spaces in support of aerenchyma. The explanation for bursting could not be resolved; it may have occurred through catalyzed lysigenous development, or it may have resulted from simple physical rupturing of primary cell walls enriched in pectin but deficient in constraining cellulose microfibrils.

Excepting a study of the helical distribution of procambial leaf primordia in the developing cortex a few mm basal to the apical meristem^[63]^, no description of how leaf traces develop and persist in *Abies* spp. could be found in the botanical literature (an exhaustive search was not possible). The development as observed in this investigation indicates that the leaf trace begins unusually, as a cortical resin duct lacking procambium or other vascular tissue. The cortical duct channel is invaded by intrusively growing axial parenchyma cells that subsequently subdivide, by repeated anticlinal and periclinal divisions, to populate the duct with well-ordered radial files of small diameter cells, most of which differentiate into woody elements having annular ribs, similar to those of the primary xylem. Before the onset of this xylogenesis, it may be that phloem translocatory cells differentiate around the perimeter of the duct; living cells are abundantly present around the developing woody duct; however, none could be definitively identified as sieve cells (see Fig. 5). Viewed in cross sections, each cortical woody duct is bisected radially by one or two radial files of enlarged thin-walled parenchyma and, as previously noted in relation to general gišogenesis, those ray cells may fulfill an essential role in signal transduction in support of vascular development^[3]^.

Earlier findings indicate that cambium formation is induced in response to the polar transport of auxin and intercellular compression^[37]^, and after a cambium forms its maintenance in terms of fusiform cell shape and length require an ongoing supply of auxin^[33]^. However, auxin also is assumed to initiate xylogenesis, but this investigation revealed that xylogenesis within cortical woody ducts occurred before, evidently independently of, subsequent formation of the woody duct's cambium. Despite the other observed effects on cellular differentiation within the cortex, no evidence was found for any of exogenous ACC, IAA, and/or IBA inducing cambium to form or altering any aspect of cortical woody duct anatomy or formation.

**Figure 9:**
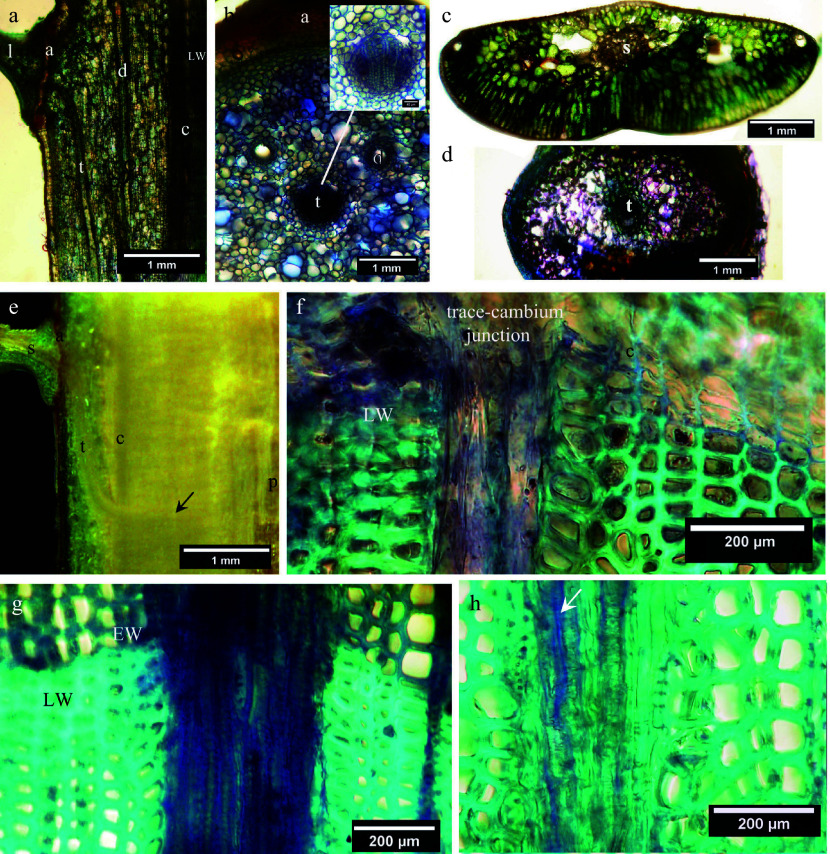
Hand-cut sections of untreated balsam-fir dormant stems: (a), (c) and (e) are unstained sections; (b), (d), (f) and (g) are stained with toluidine blue. (a) Radial section of dormant cortex showing the abscission zone (a) and axially oriented leaf trace (t) of the mature first-year leaf (l), also showing a resin duct (d), latewood (LW) and vascular cambium (c). (b) Cross section basal to the leaf abscission zone (a) of the leader showing the cortical leaf trace (t); the inset at higher magnification shows the trace as a woody duct. (c) An unstained cross section at midway along the length of a 3-year-old balsam fir needle, showing its central vascular strand (s). (d) Tangential section of a 3-year-old balsam fir needle base at its stem attachment point showing the singular leaf trace (t) entering the stem. (e) Radial section of a 3-year-old balsam fir stem showing the vascular strand in the leaf (s) passing through the abscission zone (a) into the woody duct leaf trace (t) in the periderm. The trace traverses the cambium (c) and runs radially through three annual layers of secondary xylem (arrow) to the pith (p). (f) Cross section through the trace-cambium junction of a 3-year-old balsam fir stem, with vascular cambium (c) and latewood (LW) also indicated. Note that most leaf-trace cells are radially elongated but thin walled. (g) Cross section through latewood (LW) of the second year and earlywood (EW) of the third year in a 3-year-old balsam fir stem showing the size and anatomy of the leaf trace. (h) Cross section through second year earlywood in a 3-year-old balsam fir stem; the trace comprises mostly tracheids but also has living cells (arrow) that appear to be sieve elements.

Examination of 3-year-old balsam-fir stem regions revealed that the overall anatomical structure of woody ducts in the periderm remained unchanged from that of the cortical woody duct originally produced during leader growth (Fig. 9).

While in peridermal tissue, the woody duct's cambium remains dormant, unchanged from the structure produced in the cortex. Assuming that the woody duct cambium remains capable of reactivation, its persistence as a dormant peridermal meristem may explain how preventitious buds sometimes form in *Abies* bark^[64]^.

In contrast, at the junction of the leaf trace with the vascular cambium, the evidence indicates that the woody duct cambium becomes meristematically active, followed by radial cell elongation and differentiation of its derivative cells at the trace's interface with the secondary xylem to produce xylem, phloem and parenchyma elements of the trace (Fig. 9). The continuing radial growth of the trace in pace with that of secondary xylem presumably serves to provide the mature leaf with water and nutriment, and on this basis it can be suggested that activity of the woody duct cambium may be the primary basis for the well-known multi-year longevity of *Abies* leaves. However, this interpretation of meristematic activity by the woody duct cambium at the trace-cambium junctions seems to be logically flawed on the basis that IAA when transported basipetally *via* the leaf trace is nevertheless without effect on living cells in the cortex or periderm but then promotes its growth at the trace-cambium junction. On the other hand, serial transverse sections prepared over the axial length of the woody duct leaf trace in the cortex revealed that some places were entirely lacking of tracheary elements within the duct channel, which seems to indicate variable transmission or reception of the xylogenesis signal. Interruptions in primary xylem continuity over the length of the cortical trace also indicates that conduction of water and nutriment into the leaf from the secondary xylem of the stem must be principally through living cells circumscribing the duct.

## Conclusions

This investigation revealed that isolated dormant stem segments lacking buds, leaves, and roots are competent to produce a variety of cell types in response to applications of micromolar concentrations of indole-3-acetic acid (IAA), indole-3-butyric acid (IBA), 1-aminocyclopropane-1-carboxylic acid (ACC) and combinations of those three. Those three hormones in combination appeared to mimic the overall effect of mature leaves left on phytohormone-untreated stem segments in their regulation of cambial growth, secondary phloem, and xylem development. IAA, IBA, and ACC individually yielded some xylogenesis, and ACC with IAA or IBA appeared to enhance the response.

This study provides new information about the stem cortex, its diverse cell types and their varied sizes, shapes, wall thicknesses, polarities, and probable specializations. Cortical growth responses below hormone application sites were stronger than those of vascular development. IBA promoted the early stages of cortical resin duct formation, whereas IAA promoted radial expansion of existing resin ducts, and both promoted radial elongation of parenchyma. The cortical woody duct, formation of which precedes leaf trace development and is dependent on a factor from mature leaves, is an entirely novel anatomical observation deserving further research.

## SUPPLEMENTARY DATA

Supplementary data to this article can be found online.

## Data Availability

All data generated or analyzed during this study are included in this published article and its supplementary information files. References 32, 37, 43 and 49 lack a doi but are available at www.researchgate.net/profile/Rodney-Savidge.
