# Supplementary material for: Responses of isolated balsam-fir stem segments to exogenous ACC, IAA, and IBA
Source: For Res (Fayettev). 2024 Sep 30;4:e033. doi: 10.48130/forres-0024-0030 (PMC11524308; doi:10.48130/forres-0024-0030)
Supplement: Supplementary file 1 — Supplementary data to this article can be found online. [file forres-0024-0030-S1.zip › 10.48130_forres-0024-0030-Suppl-FigureS1.pdf]

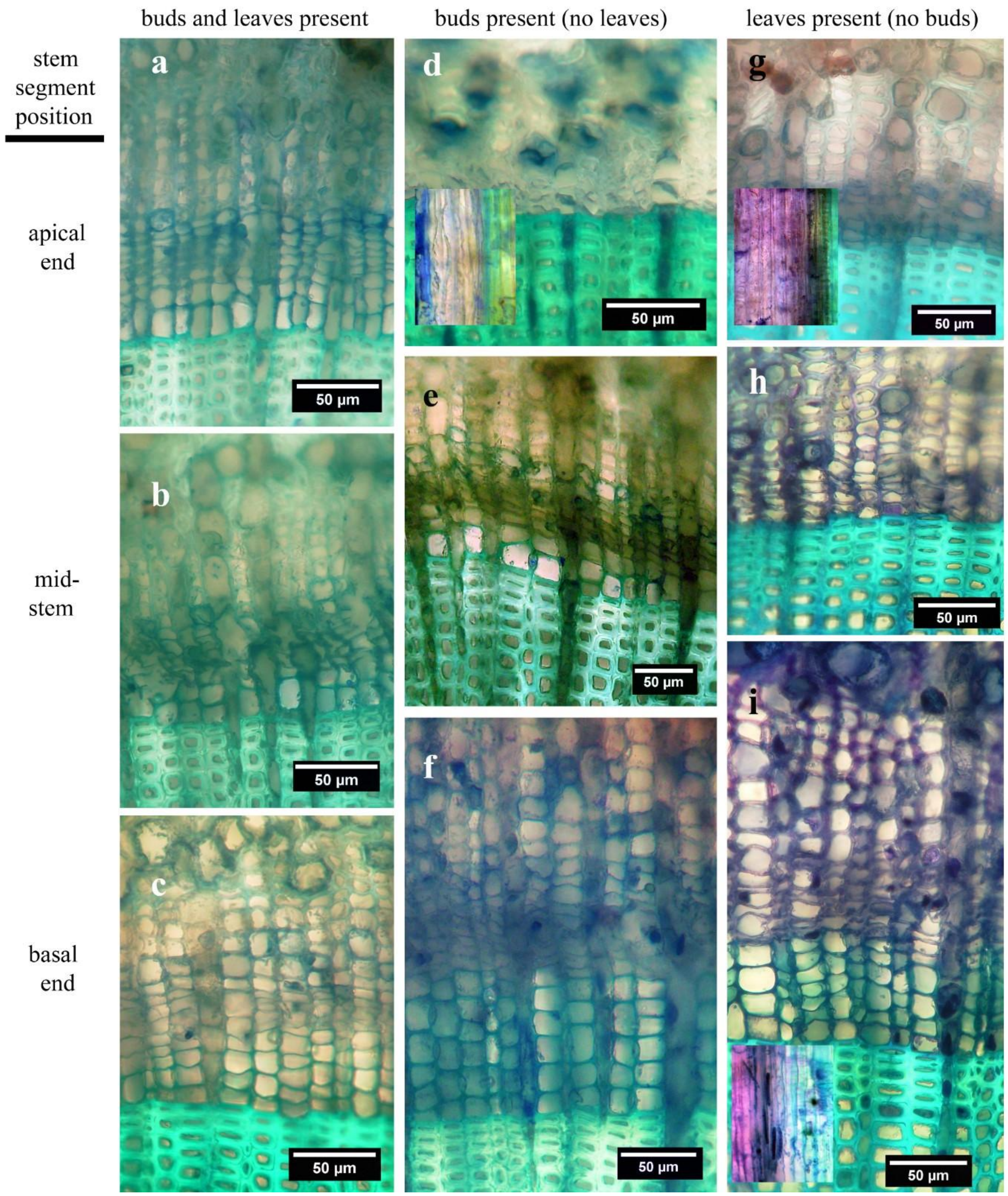

Supplementary figure S1. Examples of the responses as they appeared on March 19th of stem segments having buds and/or leaves and not treated with a phytohormone. (a)−(c) Apical, mid-stem and basal regions, respectively, of segments having both buds and leaves present. (d)−(f) Apical, mid-stem and basal regions, respectively, of segment having only a whorl of terminal buds present. (g)−(i) Apical, mid-stem and basal regions of a segment having only leaves present. The inset images in (d), (g) and (i) show the appearance of the cambial zone and adjoining cells at those positions as they appeared in radial section.
